# Supplementary material for: Transcriptome Analysis Reveals Vimentin-Induced Disruption of Cell–Cell Associations Augments Breast Cancer Cell Migration
Source: Cells. 2022 Dec 13;11(24):4035. doi: 10.3390/cells11244035 (PMC9776984; doi:10.3390/cells11244035)
Supplement: Supplementary file 1 [file cells-11-04035-s001.zip › DEGs and enrichment analysis.zip!/DEGs and enrichment analysis/WT_VIMvsVector KEGG/GSEA/WT_VIM/SPLICEOSOME/SPLICEOSOME(HSA03040).html]

Details for gene set SPLICEOSOME(HSA03040)[GSEA]

|  || Dataset | fpkm.sample |
| Phenotype | sample.cls |
| Upregulated in class | WT\_VIM |
| GeneSet | SPLICEOSOME(HSA03040) |
| Enrichment Score (ES) | 0.6374656 |
| Normalized Enrichment Score (NES) | 1.1557457 |
| Nominal p-value | 0.0 |
| FDR q-value | 0.2873328 |
| FWER p-Value | 0.498 |
Table: GSEA Results Summary

  

Fig 1: Enrichment plot: SPLICEOSOME(HSA03040)      
 Profile of the Running ES Score & Positions of GeneSet Members on the Rank Ordered List

  

| PROBE | DESCRIPTION (from dataset) | GENE SYMBOL | GENE\_TITLE | RANK IN GENE LIST | RANK METRIC SCORE | RUNNING ES | CORE ENRICHMENT || 1 | ENSG00000217835 | AL034397.1 |  |  | 120 | Infinity | -0.0020 | Yes |
| 2 | ENSG00000225787 | LSM3P3 |  |  | 590 | Infinity | -0.0100 | Yes |
| 3 | ENSG00000230299 | AC093162.1 |  |  | 776 | Infinity | -0.0131 | Yes |
| 4 | ENSG00000229738 | AC010240.1 |  |  | 789 | Infinity | -0.0133 | Yes |
| 5 | ENSG00000244730 | AC139451.1 |  |  | 874 | Infinity | -0.0147 | Yes |
| 6 | ENSG00000225642 | SNRPEP5 |  |  | 1181 | Infinity | -0.0199 | Yes |
| 7 | ENSG00000264350 | SNRPGP2 |  |  | 1436 | Infinity | -0.0242 | Yes |
| 8 | ENSG00000235763 | SNRPGP5 |  |  | 1478 | Infinity | -0.0248 | Yes |
| 9 | ENSG00000170089 | AC106795.1 |  |  | 1536 | Infinity | -0.0258 | Yes |
| 10 | ENSG00000250662 | HNRNPKP5 |  |  | 1540 | Infinity | -0.0258 | Yes |
| 11 | ENSG00000229661 | BUD31P2 |  |  | 1573 | Infinity | -0.0263 | Yes |
| 12 | ENSG00000253223 | AC110998.1 |  |  | 1661 | Infinity | -0.0278 | Yes |
| 13 | ENSG00000213972 | AL024509.1 |  |  | 1899 | Infinity | -0.0318 | Yes |
| 14 | ENSG00000259512 | HNRNPA1P5 |  |  | 1901 | Infinity | -0.0318 | Yes |
| 15 | ENSG00000226790 | HNRNPA3P1 |  |  | 2111 | Infinity | -0.0353 | Yes |
| 16 | ENSG00000249465 | RBMXP4 |  |  | 2246 | Infinity | -0.0376 | Yes |
| 17 | ENSG00000214761 | HNRNPA1P15 |  |  | 2309 | Infinity | -0.0386 | Yes |
| 18 | ENSG00000259931 | AC105036.1 |  |  | 2310 | Infinity | -0.0386 | Yes |
| 19 | ENSG00000275929 | AC068831.5 |  |  | 2380 | Infinity | -0.0397 | Yes |
| 20 | ENSG00000228020 | HNRNPA1P46 |  |  | 2464 | 13.913 | -0.0028 | Yes |
| 21 | ENSG00000228366 | AL592437.1 |  |  | 2531 | 9.958 | 0.0234 | Yes |
| 22 | ENSG00000229251 | HNRNPA1P8 |  |  | 2601 | 8.085 | 0.0445 | Yes |
| 23 | ENSG00000256356 | HSPA8P5 |  |  | 2616 | 7.882 | 0.0660 | Yes |
| 24 | ENSG00000232953 | HSPA8P18 |  |  | 2631 | 7.706 | 0.0869 | Yes |
| 25 | ENSG00000213683 | AC002056.1 |  |  | 2694 | 6.942 | 0.1050 | Yes |
| 26 | ENSG00000204389 | HSPA1A |  |  | 2920 | 5.273 | 0.1157 | Yes |
| 27 | ENSG00000235363 | SNRPGP10 |  |  | 3100 | 4.554 | 0.1252 | Yes |
| 28 | novel.574 | - |  |  | 3113 | 4.473 | 0.1373 | Yes |
| 29 | novel.590 | - |  |  | 3133 | 4.425 | 0.1491 | Yes |
| 30 | ENSG00000258427 | RBM8B |  |  | 3138 | 4.406 | 0.1612 | Yes |
| 31 | ENSG00000220937 | HNRNPA1P41 |  |  | 3237 | 4.146 | 0.1709 | Yes |
| 32 | ENSG00000244668 | SNRPCP3 |  |  | 3259 | 4.049 | 0.1817 | Yes |
| 33 | ENSG00000251235 | SNRPCP2 |  |  | 3335 | 3.955 | 0.1913 | Yes |
| 34 | ENSG00000225695 | HNRNPA1P35 |  |  | 3359 | 3.854 | 0.2016 | Yes |
| 35 | ENSG00000254449 | SF3A3P2 |  |  | 3460 | 3.690 | 0.2100 | Yes |
| 36 | ENSG00000227347 | HNRNPKP2 |  |  | 3645 | 3.435 | 0.2163 | Yes |
| 37 | ENSG00000235554 | AC005822.1 |  |  | 3802 | 3.274 | 0.2227 | Yes |
| 38 | ENSG00000237017 | AC245052.4 |  |  | 3887 | 3.149 | 0.2299 | Yes |
| 39 | ENSG00000130520 | LSM4 |  |  | 4018 | 3.001 | 0.2360 | Yes |
| 40 | ENSG00000259707 | AC105339.4 |  |  | 4046 | 2.987 | 0.2437 | Yes |
| 41 | ENSG00000130332 | LSM7 |  |  | 4112 | 2.945 | 0.2507 | Yes |
| 42 | ENSG00000169564 | PCBP1 |  |  | 4159 | 2.891 | 0.2579 | Yes |
| 43 | ENSG00000232499 | AL391058.1 |  |  | 4269 | 2.807 | 0.2638 | Yes |
| 44 | ENSG00000169976 | SF3B5 |  |  | 4282 | 2.798 | 0.2713 | Yes |
| 45 | ENSG00000179950 | PUF60 |  |  | 4291 | 2.789 | 0.2788 | Yes |
| 46 | ENSG00000230911 | PPIHP1 |  |  | 4342 | 2.774 | 0.2856 | Yes |
| 47 | ENSG00000183684 | ALYREF |  |  | 4366 | 2.756 | 0.2928 | Yes |
| 48 | ENSG00000077312 | SNRPA |  |  | 4407 | 2.722 | 0.2996 | Yes |
| 49 | ENSG00000270553 | AC011921.3 |  |  | 4432 | 2.698 | 0.3066 | Yes |
| 50 | ENSG00000219102 | HNRNPA3P12 |  |  | 4500 | 2.648 | 0.3128 | Yes |
| 51 | ENSG00000174231 | PRPF8 |  |  | 4546 | 2.637 | 0.3192 | Yes |
| 52 | ENSG00000076924 | XAB2 |  |  | 4744 | 2.531 | 0.3229 | Yes |
| 53 | ENSG00000215210 | RBMXP2 |  |  | 5091 | 2.355 | 0.3234 | Yes |
| 54 | ENSG00000257195 | HNRNPA1P50 |  |  | 5188 | 2.314 | 0.3282 | Yes |
| 55 | ENSG00000125835 | SNRPB |  |  | 5251 | 2.301 | 0.3335 | Yes |
| 56 | ENSG00000204253 | HNRNPCP2 |  |  | 5361 | 2.249 | 0.3378 | Yes |
| 57 | ENSG00000234332 | BCAS2P2 |  |  | 5438 | 2.219 | 0.3426 | Yes |
| 58 | ENSG00000224773 | HSPA8P7 |  |  | 5604 | 2.159 | 0.3457 | Yes |
| 59 | novel.610 | - |  |  | 5630 | 2.159 | 0.3512 | Yes |
| 60 | ENSG00000125743 | SNRPD2 |  |  | 5671 | 2.146 | 0.3565 | Yes |
| 61 | ENSG00000214653 | HNRNPA3P3 |  |  | 5820 | 2.090 | 0.3597 | Yes |
| 62 | ENSG00000137168 | PPIL1 |  |  | 5862 | 2.076 | 0.3647 | Yes |
| 63 | ENSG00000100028 | SNRPD3 |  |  | 5906 | 2.061 | 0.3697 | Yes |
| 64 | ENSG00000060688 | SNRNP40 |  |  | 5959 | 2.045 | 0.3744 | Yes |
| 65 | ENSG00000110107 | PRPF19 |  |  | 6001 | 2.033 | 0.3793 | Yes |
| 66 | ENSG00000085872 | CHERP |  |  | 6022 | 2.028 | 0.3845 | Yes |
| 67 | ENSG00000224578 | HNRNPA1P48 |  |  | 6059 | 2.015 | 0.3895 | Yes |
| 68 | ENSG00000251484 | AL356488.1 |  |  | 6136 | 1.993 | 0.3937 | Yes |
| 69 | ENSG00000236478 | AC012513.2 |  |  | 6201 | 1.992 | 0.3981 | Yes |
| 70 | ENSG00000101161 | PRPF6 |  |  | 6355 | 1.960 | 0.4008 | Yes |
| 71 | ENSG00000140829 | DHX38 |  |  | 6358 | 1.959 | 0.4062 | Yes |
| 72 | ENSG00000141543 | EIF4A3 |  |  | 6390 | 1.950 | 0.4110 | Yes |
| 73 | ENSG00000189091 | SF3B3 |  |  | 6449 | 1.935 | 0.4154 | Yes |
| 74 | ENSG00000250859 | HNRNPKP1 |  |  | 6568 | 1.909 | 0.4186 | Yes |
| 75 | ENSG00000063244 | U2AF2 |  |  | 6653 | 1.888 | 0.4224 | Yes |
| 76 | ENSG00000160799 | CCDC12 |  |  | 6669 | 1.884 | 0.4273 | Yes |
| 77 | ENSG00000175467 | SART1 |  |  | 6689 | 1.881 | 0.4322 | Yes |
| 78 | novel.430 | - |  |  | 6723 | 1.872 | 0.4368 | Yes |
| 79 | ENSG00000111786 | SRSF9 |  |  | 6734 | 1.867 | 0.4417 | Yes |
| 80 | ENSG00000260653 | AC237221.1 |  |  | 6761 | 1.863 | 0.4464 | Yes |
| 81 | ENSG00000205763 | RP9P |  |  | 6844 | 1.856 | 0.4501 | Yes |
| 82 | ENSG00000143368 | SF3B4 |  |  | 6896 | 1.842 | 0.4543 | Yes |
| 83 | ENSG00000231878 | SNRPFP1 |  |  | 7152 | 1.784 | 0.4549 | Yes |
| 84 | ENSG00000279069 | AC015813.5 |  |  | 7317 | 1.753 | 0.4569 | Yes |
| 85 | ENSG00000100410 | PHF5A |  |  | 7375 | 1.741 | 0.4607 | Yes |
| 86 | ENSG00000100138 | SNU13 |  |  | 7572 | 1.701 | 0.4621 | Yes |
| 87 | ENSG00000171960 | PPIH |  |  | 7582 | 1.698 | 0.4666 | Yes |
| 88 | ENSG00000108883 | EFTUD2 |  |  | 7620 | 1.692 | 0.4706 | Yes |
| 89 | ENSG00000104897 | SF3A2 |  |  | 7656 | 1.684 | 0.4747 | Yes |
| 90 | ENSG00000099783 | HNRNPM |  |  | 7667 | 1.683 | 0.4791 | Yes |
| 91 | ENSG00000144028 | SNRNP200 |  |  | 7677 | 1.681 | 0.4836 | Yes |
| 92 | ENSG00000124562 | SNRPC |  |  | 7735 | 1.669 | 0.4872 | Yes |
| 93 | ENSG00000259462 | CPEB1-AS1 |  |  | 7851 | 1.649 | 0.4898 | Yes |
| 94 | ENSG00000141759 | TXNL4A |  |  | 7888 | 1.643 | 0.4937 | Yes |
| 95 | ENSG00000126803 | HSPA2 |  |  | 7911 | 1.639 | 0.4979 | Yes |
| 96 | ENSG00000219395 | HSPA8P15 |  |  | 7945 | 1.632 | 0.5018 | Yes |
| 97 | ENSG00000144158 | AL078621.1 |  |  | 7972 | 1.627 | 0.5058 | Yes |
| 98 | ENSG00000233270 | SNRPEP4 |  |  | 8027 | 1.619 | 0.5094 | Yes |
| 99 | ENSG00000182004 | SNRPE |  |  | 8186 | 1.597 | 0.5111 | Yes |
| 100 | ENSG00000139343 | SNRPF |  |  | 8237 | 1.587 | 0.5146 | Yes |
| 101 | ENSG00000257851 | HNRNPA3P10 |  |  | 8450 | 1.553 | 0.5152 | Yes |
| 102 | ENSG00000136875 | PRPF4 |  |  | 8591 | 1.536 | 0.5171 | Yes |
| 103 | ENSG00000143977 | SNRPG |  |  | 8647 | 1.528 | 0.5204 | Yes |
| 104 | novel.611 | - |  |  | 8764 | 1.512 | 0.5225 | Yes |
| 105 | ENSG00000102103 | PQBP1 |  |  | 9010 | 1.480 | 0.5224 | Yes |
| 106 | ENSG00000214223 | HNRNPA1P10 |  |  | 9011 | 1.479 | 0.5265 | Yes |
| 107 | ENSG00000051596 | THOC3 |  |  | 9077 | 1.470 | 0.5294 | Yes |
| 108 | ENSG00000084463 | WBP11 |  |  | 9161 | 1.459 | 0.5320 | Yes |
| 109 | ENSG00000236565 | HNRNPA3P5 |  |  | 9200 | 1.453 | 0.5354 | Yes |
| 110 | ENSG00000092199 | HNRNPC |  |  | 9266 | 1.443 | 0.5383 | Yes |
| 111 | ENSG00000084072 | PPIE |  |  | 9351 | 1.436 | 0.5408 | Yes |
| 112 | ENSG00000131876 | SNRPA1 |  |  | 9373 | 1.434 | 0.5444 | Yes |
| 113 | ENSG00000168883 | USP39 |  |  | 9442 | 1.424 | 0.5471 | Yes |
| 114 | ENSG00000256968 | SNRPEP2 |  |  | 9536 | 1.412 | 0.5494 | Yes |
| 115 | ENSG00000174243 | DDX23 |  |  | 9561 | 1.409 | 0.5529 | Yes |
| 116 | ENSG00000111196 | MAGOHB |  |  | 9589 | 1.406 | 0.5563 | Yes |
| 117 | ENSG00000106355 | LSM5 |  |  | 9599 | 1.405 | 0.5600 | Yes |
| 118 | ENSG00000099995 | SF3A1 |  |  | 9875 | 1.374 | 0.5591 | Yes |
| 119 | ENSG00000160201 | U2AF1 |  |  | 9979 | 1.362 | 0.5611 | Yes |
| 120 | ENSG00000067596 | DHX8 |  |  | 10300 | 1.325 | 0.5593 | Yes |
| 121 | ENSG00000275895 | U2AF1L5 |  |  | 10356 | 1.320 | 0.5620 | Yes |
| 122 | ENSG00000226068 | HNRNPA3P4 |  |  | 10490 | 1.318 | 0.5634 | Yes |
| 123 | novel.584 | - |  |  | 10567 | 1.309 | 0.5657 | Yes |
| 124 | ENSG00000115875 | SRSF7 |  |  | 10594 | 1.306 | 0.5688 | Yes |
| 125 | ENSG00000087365 | SF3B2 |  |  | 10759 | 1.288 | 0.5696 | Yes |
| 126 | ENSG00000183431 | SF3A3 |  |  | 10791 | 1.285 | 0.5726 | Yes |
| 127 | ENSG00000213300 | HNRNPA3P6 |  |  | 10858 | 1.278 | 0.5750 | Yes |
| 128 | ENSG00000257043 | AP006296.1 |  |  | 11027 | 1.262 | 0.5756 | Yes |
| 129 | ENSG00000128534 | LSM8 |  |  | 11200 | 1.247 | 0.5761 | Yes |
| 130 | ENSG00000132792 | CTNNBL1 |  |  | 11244 | 1.243 | 0.5788 | Yes |
| 131 | ENSG00000104852 | SNRNP70 |  |  | 11261 | 1.241 | 0.5819 | Yes |
| 132 | ENSG00000243547 | HNRNPKP4 |  |  | 11315 | 1.236 | 0.5844 | Yes |
| 133 | ENSG00000259397 | AC021231.2 |  |  | 11375 | 1.230 | 0.5868 | Yes |
| 134 | ENSG00000262477 | AC021224.1 |  |  | 11449 | 1.230 | 0.5889 | Yes |
| 135 | ENSG00000170860 | LSM3 |  |  | 11731 | 1.207 | 0.5875 | Yes |
| 136 | ENSG00000109971 | HSPA8 |  |  | 11736 | 1.206 | 0.5907 | Yes |
| 137 | ENSG00000164610 | RP9 |  |  | 11743 | 1.206 | 0.5940 | Yes |
| 138 | ENSG00000136527 | TRA2B |  |  | 12003 | 1.181 | 0.5928 | Yes |
| 139 | ENSG00000115128 | SF3B6 |  |  | 12066 | 1.176 | 0.5950 | Yes |
| 140 | ENSG00000173110 | HSPA6 |  |  | 12149 | 1.169 | 0.5968 | Yes |
| 141 | ENSG00000167088 | SNRPD1 |  |  | 12227 | 1.162 | 0.5987 | Yes |
| 142 | ENSG00000161547 | SRSF2 |  |  | 12475 | 1.143 | 0.5976 | Yes |
| 143 | ENSG00000265241 | RBM8A |  |  | 12506 | 1.140 | 0.6002 | Yes |
| 144 | ENSG00000135486 | HNRNPA1 |  |  | 12524 | 1.138 | 0.6031 | Yes |
| 145 | ENSG00000262333 | HNRNPA1P16 |  |  | 12610 | 1.131 | 0.6048 | Yes |
| 146 | ENSG00000164167 | LSM6 |  |  | 12659 | 1.127 | 0.6070 | Yes |
| 147 | ENSG00000109606 | DHX15 |  |  | 12974 | 1.103 | 0.6047 | Yes |
| 148 | ENSG00000279827 | AC136469.2 |  |  | 12976 | 1.103 | 0.6077 | Yes |
| 149 | ENSG00000263465 | SRSF8 |  |  | 13187 | 1.089 | 0.6072 | Yes |
| 150 | ENSG00000153187 | HNRNPU |  |  | 13227 | 1.085 | 0.6095 | Yes |
| 151 | ENSG00000113649 | TCERG1 |  |  | 13281 | 1.080 | 0.6116 | Yes |
| 152 | ENSG00000136937 | NCBP1 |  |  | 13301 | 1.079 | 0.6142 | Yes |
| 153 | ENSG00000112081 | SRSF3 |  |  | 13324 | 1.078 | 0.6168 | Yes |
| 154 | ENSG00000134748 | PRPF38A |  |  | 13373 | 1.074 | 0.6189 | Yes |
| 155 | ENSG00000139675 | HNRNPA1L2 |  |  | 13385 | 1.073 | 0.6217 | Yes |
| 156 | ENSG00000259051 | HNRNPUP1 |  |  | 13426 | 1.069 | 0.6240 | Yes |
| 157 | ENSG00000215492 | HNRNPA1P7 |  |  | 13461 | 1.067 | 0.6263 | Yes |
| 158 | ENSG00000105618 | PRPF31 |  |  | 13474 | 1.066 | 0.6290 | Yes |
| 159 | ENSG00000213516 | RBMXL1 |  |  | 13705 | 1.049 | 0.6280 | Yes |
| 160 | ENSG00000021776 | AQR |  |  | 13974 | 1.028 | 0.6263 | Yes |
| 161 | ENSG00000124193 | SRSF6 |  |  | 14190 | 1.011 | 0.6254 | Yes |
| 162 | ENSG00000206228 | HNRNPA1P4 |  |  | 14294 | 1.004 | 0.6264 | Yes |
| 163 | ENSG00000116752 | BCAS2 |  |  | 14339 | 1.000 | 0.6284 | Yes |
| 164 | ENSG00000145833 | DDX46 |  |  | 14345 | 0.999 | 0.6311 | Yes |
| 165 | ENSG00000124380 | SNRNP27 |  |  | 14851 | 0.979 | 0.6252 | Yes |
| 166 | ENSG00000165119 | HNRNPK |  |  | 14914 | 0.975 | 0.6268 | Yes |
| 167 | ENSG00000096401 | CDC5L |  |  | 15043 | 0.968 | 0.6273 | Yes |
| 168 | ENSG00000117360 | PRPF3 |  |  | 15171 | 0.961 | 0.6277 | Yes |
| 169 | ENSG00000100813 | ACIN1 |  |  | 15282 | 0.953 | 0.6285 | Yes |
| 170 | ENSG00000162385 | MAGOH |  |  | 15420 | 0.945 | 0.6288 | Yes |
| 171 | ENSG00000134453 | RBM17 |  |  | 15624 | 0.931 | 0.6279 | Yes |
| 172 | novel.666 | - |  |  | 15802 | 0.929 | 0.6274 | Yes |
| 173 | ENSG00000101343 | CRNKL1 |  |  | 15980 | 0.921 | 0.6269 | Yes |
| 174 | ENSG00000161265 | U2AF1L4 |  |  | 16016 | 0.918 | 0.6289 | Yes |
| 175 | ENSG00000136450 | SRSF1 |  |  | 16150 | 0.910 | 0.6291 | Yes |
| 176 | ENSG00000168438 | CDC40 |  |  | 16534 | 0.886 | 0.6250 | Yes |
| 177 | ENSG00000196504 | PRPF40A |  |  | 16596 | 0.882 | 0.6264 | Yes |
| 178 | ENSG00000198231 | DDX42 |  |  | 16703 | 0.876 | 0.6270 | Yes |
| 179 | ENSG00000116350 | SRSF4 |  |  | 16732 | 0.874 | 0.6289 | Yes |
| 180 | ENSG00000170144 | HNRNPA3 |  |  | 16786 | 0.870 | 0.6304 | Yes |
| 181 | ENSG00000146007 | ZMAT2 |  |  | 16791 | 0.870 | 0.6327 | Yes |
| 182 | ENSG00000114503 | NCBP2 |  |  | 16846 | 0.867 | 0.6342 | Yes |
| 183 | ENSG00000188529 | SRSF10 |  |  | 16928 | 0.861 | 0.6352 | Yes |
| 184 | ENSG00000150316 | CWC15 |  |  | 16935 | 0.861 | 0.6375 | Yes |
| 185 | ENSG00000079134 | THOC1 |  |  | 17200 | 0.845 | 0.6353 | No |
| 186 | ENSG00000108654 | DDX5 |  |  | 17263 | 0.841 | 0.6365 | No |
| 187 | ENSG00000163714 | U2SURP |  |  | 17478 | 0.829 | 0.6352 | No |
| 188 | ENSG00000171566 | PLRG1 |  |  | 17546 | 0.824 | 0.6363 | No |
| 189 | ENSG00000240682 | ISY1 |  |  | 17721 | 0.815 | 0.6356 | No |
| 190 | ENSG00000086589 | RBM22 |  |  | 18081 | 0.795 | 0.6317 | No |
| 191 | ENSG00000115524 | SF3B1 |  |  | 18153 | 0.790 | 0.6326 | No |
| 192 | ENSG00000100603 | SNW1 |  |  | 18682 | 0.761 | 0.6257 | No |
| 193 | ENSG00000100650 | SRSF5 |  |  | 18770 | 0.755 | 0.6263 | No |
| 194 | ENSG00000225578 | NCBP2-AS1 |  |  | 18796 | 0.753 | 0.6280 | No |
| 195 | ENSG00000106245 | BUD31 |  |  | 18989 | 0.743 | 0.6267 | No |
| 196 | ENSG00000164548 | TRA2A |  |  | 19004 | 0.742 | 0.6285 | No |
| 197 | ENSG00000125870 | SNRPB2 |  |  | 19150 | 0.734 | 0.6281 | No |
| 198 | ENSG00000270903 | HNRNPA3P9 |  |  | 19178 | 0.734 | 0.6296 | No |
| 199 | ENSG00000225217 | HSPA7 |  |  | 19777 | 0.701 | 0.6214 | No |
| 200 | ENSG00000233231 | HNRNPA1P49 |  |  | 20042 | 0.686 | 0.6188 | No |
| 201 | ENSG00000119953 | SMNDC1 |  |  | 20150 | 0.680 | 0.6188 | No |
| 202 | ENSG00000134186 | PRPF38B |  |  | 20807 | 0.645 | 0.6094 | No |
| 203 | ENSG00000119707 | RBM25 |  |  | 20857 | 0.643 | 0.6104 | No |
| 204 | ENSG00000233514 | AL356653.1 |  |  | 21003 | 0.636 | 0.6096 | No |
| 205 | ENSG00000224543 | SNRPGP15 |  |  | 21101 | 0.632 | 0.6097 | No |
| 206 | ENSG00000164609 | SLU7 |  |  | 21253 | 0.622 | 0.6089 | No |
| 207 | novel.655 | - |  |  | 21345 | 0.617 | 0.6090 | No |
| 208 | ENSG00000125676 | THOC2 |  |  | 21459 | 0.612 | 0.6088 | No |
| 209 | ENSG00000227638 | HNRNPA1P14 |  |  | 22067 | 0.573 | 0.6000 | No |
| 210 | ENSG00000228653 | HNRNPCP7 |  |  | 22145 | 0.567 | 0.6003 | No |
| 211 | ENSG00000231942 | HNRNPA1P36 |  |  | 22178 | 0.565 | 0.6013 | No |
| 212 | ENSG00000117614 | SYF2 |  |  | 22235 | 0.561 | 0.6019 | No |
| 213 | ENSG00000265298 | AC132812.1 |  |  | 22437 | 0.549 | 0.5999 | No |
| 214 | ENSG00000165630 | PRPF18 |  |  | 22571 | 0.539 | 0.5992 | No |
| 215 | ENSG00000220157 | HNRNPA1P12 |  |  | 22808 | 0.520 | 0.5966 | No |
| 216 | novel.583 | - |  |  | 23084 | 0.500 | 0.5933 | No |
| 217 | ENSG00000258900 | HNRNPCP1 |  |  | 23092 | 0.499 | 0.5945 | No |
| 218 | ENSG00000213411 | RBM22P2 |  |  | 23164 | 0.498 | 0.5947 | No |
| 219 | ENSG00000230280 | HNRNPA1P59 |  |  | 23648 | 0.466 | 0.5877 | No |
| 220 | ENSG00000243970 | PPIEL |  |  | 23925 | 0.444 | 0.5842 | No |
| 221 | ENSG00000230568 | SF3A3P1 |  |  | 24946 | 0.359 | 0.5678 | No |
| 222 | ENSG00000214867 | SRSF9P1 |  |  | 25083 | 0.342 | 0.5665 | No |
| 223 | ENSG00000259411 | HNRNPA1P45 |  |  | 25178 | 0.332 | 0.5658 | No |
| 224 | ENSG00000267717 | SRSF10P1 |  |  | 25198 | 0.330 | 0.5664 | No |
| 225 | ENSG00000267203 | SNRPGP4 |  |  | 25797 | 0.271 | 0.5569 | No |
| 226 | ENSG00000232296 | AL691482.1 |  |  | 26447 | 0.196 | 0.5464 | No |
| 227 | ENSG00000204388 | HSPA1B |  |  | 26508 | 0.191 | 0.5459 | No |
| 228 | ENSG00000249977 | AC106748.1 |  |  | 26563 | 0.185 | 0.5455 | No |
| 229 | ENSG00000259419 | HNRNPCP3 |  |  | 27604 | 0.000 | 0.5278 | No |
| 230 | ENSG00000253119 | HNRNPA3P7 |  |  | 27661 | 0.000 | 0.5268 | No |
| 231 | ENSG00000259585 | RBM17P4 |  |  | 27741 | 0.000 | 0.5255 | No |
| 232 | ENSG00000273997 | AL355365.1 |  |  | 27883 | 0.000 | 0.5231 | No |
| 233 | ENSG00000236569 | HNRNPA1P73 |  |  | 27987 | 0.000 | 0.5213 | No |
| 234 | ENSG00000270975 | MAGOH3P |  |  | 28019 | 0.000 | 0.5208 | No |
| 235 | ENSG00000237848 | AL606489.2 |  |  | 28211 | 0.000 | 0.5175 | No |
| 236 | ENSG00000219487 | AL603766.1 |  |  | 28289 | 0.000 | 0.5162 | No |
| 237 | ENSG00000213412 | HNRNPA1P33 |  |  | 28666 | 0.000 | 0.5098 | No |
| 238 | ENSG00000238259 | AC067940.1 |  |  | 28874 | 0.000 | 0.5063 | No |
| 239 | ENSG00000271063 | SNRPGP17 |  |  | 29069 | 0.000 | 0.5030 | No |
| 240 | ENSG00000271509 | AC018714.1 |  |  | 29083 | 0.000 | 0.5027 | No |
| 241 | ENSG00000260689 | HNRNPA3P11 |  |  | 29825 | 0.000 | 0.4901 | No |
| 242 | ENSG00000227709 | AC091607.1 |  |  | 29856 | 0.000 | 0.4896 | No |
| 243 | ENSG00000248610 | HSPA8P4 |  |  | 29897 | 0.000 | 0.4889 | No |
| 244 | ENSG00000264176 | MAGOH2P |  |  | 30085 | 0.000 | 0.4857 | No |
| 245 | ENSG00000213559 | HNRNPA1P64 |  |  | 30358 | 0.000 | 0.4811 | No |
| 246 | ENSG00000225636 | HNRNPA3P15 |  |  | 30399 | 0.000 | 0.4804 | No |
| 247 | ENSG00000254079 | AC012339.2 |  |  | 30583 | 0.000 | 0.4773 | No |
| 248 | ENSG00000235627 | SNRPFP4 |  |  | 30602 | 0.000 | 0.4770 | No |
| 249 | ENSG00000254406 | AP001994.1 |  |  | 30659 | 0.000 | 0.4760 | No |
| 250 | ENSG00000249540 | AC117383.2 |  |  | 30782 | 0.000 | 0.4740 | No |
| 251 | ENSG00000237285 | HNRNPA1P2 |  |  | 30997 | NaN | 0.4703 | No |
| 252 | ENSG00000254713 | HNRNPA1P72 |  |  | 31278 | NaN | 0.4656 | No |
| 253 | ENSG00000224061 | AC092106.1 |  |  | 31433 | NaN | 0.4630 | No |
| 254 | ENSG00000239345 | HNRNPA1P26 |  |  | 31535 | NaN | 0.4613 | No |
| 255 | ENSG00000283117 | AC004949.1 |  |  | 31614 | NaN | 0.4600 | No |
| 256 | ENSG00000270447 | AC007179.3 |  |  | 31776 | NaN | 0.4573 | No |
| 257 | ENSG00000257169 | AC125612.1 |  |  | 32129 | NaN | 0.4513 | No |
| 258 | ENSG00000227862 | HNRNPA1P31 |  |  | 32192 | NaN | 0.4503 | No |
| 259 | ENSG00000230170 | HNRNPA1P28 |  |  | 32246 | NaN | 0.4494 | No |
| 260 | ENSG00000235325 | AC009963.3 |  |  | 32399 | NaN | 0.4469 | No |
| 261 | ENSG00000223982 | SNRPD2P2 |  |  | 32466 | NaN | 0.4458 | No |
| 262 | ENSG00000248640 | HNRNPA1P56 |  |  | 32683 | NaN | 0.4421 | No |
| 263 | ENSG00000241478 | HSPA8P9 |  |  | 32734 | NaN | 0.4413 | No |
| 264 | ENSG00000255063 | AP001646.2 |  |  | 32939 | NaN | 0.4378 | No |
| 265 | ENSG00000270431 | AC005000.2 |  |  | 32981 | NaN | 0.4372 | No |
| 266 | ENSG00000232048 | HNRNPCP9 |  |  | 33388 | NaN | 0.4303 | No |
| 267 | ENSG00000226734 | SNRPGP12 |  |  | 33401 | NaN | 0.4301 | No |
| 268 | ENSG00000215586 | AL031679.1 |  |  | 33474 | NaN | 0.4289 | No |
| 269 | ENSG00000248850 | AC078867.1 |  |  | 33772 | NaN | 0.4239 | No |
| 270 | ENSG00000226485 | RBM22P3 |  |  | 33951 | NaN | 0.4208 | No |
| 271 | ENSG00000241537 | AC134050.1 |  |  | 34094 | NaN | 0.4185 | No |
| 272 | ENSG00000239919 | SNRPGP3 |  |  | 34263 | NaN | 0.4156 | No |
| 273 | ENSG00000234564 | HSPA8P20 |  |  | 34314 | NaN | 0.4148 | No |
| 274 | ENSG00000226670 | BCAS2P3 |  |  | 34335 | NaN | 0.4145 | No |
| 275 | ENSG00000259586 | AC015712.3 |  |  | 34589 | NaN | 0.4102 | No |
| 276 | ENSG00000237442 | HNRNPA1P57 |  |  | 34662 | NaN | 0.4090 | No |
| 277 | ENSG00000251126 | AC017007.5 |  |  | 34722 | NaN | 0.4080 | No |
| 278 | ENSG00000198563 | DDX39B |  |  | 34746 | NaN | 0.4077 | No |
| 279 | ENSG00000229910 | HNRNPA1P18 |  |  | 34762 | NaN | 0.4074 | No |
| 280 | ENSG00000213305 | HNRNPCP6 |  |  | 34914 | NaN | 0.4049 | No |
| 281 | ENSG00000230727 | RBMY2WP |  |  | 34999 | NaN | 0.4035 | No |
| 282 | ENSG00000229887 | HNRNPA1P6 |  |  | 35199 | NaN | 0.4001 | No |
| 283 | ENSG00000215286 | AL031584.1 |  |  | 35244 | NaN | 0.3994 | No |
| 284 | ENSG00000254748 | HNRNPCP8 |  |  | 35330 | NaN | 0.3980 | No |
| 285 | ENSG00000266181 | EIF4A3P1 |  |  | 35430 | NaN | 0.3963 | No |
| 286 | ENSG00000255141 | HNRNPA1P76 |  |  | 35463 | NaN | 0.3958 | No |
| 287 | ENSG00000229906 | SNRPGP11 |  |  | 35485 | NaN | 0.3955 | No |
| 288 | ENSG00000230946 | HNRNPA1P68 |  |  | 35497 | NaN | 0.3953 | No |
| 289 | ENSG00000273999 | RBM17P2 |  |  | 35831 | NaN | 0.3897 | No |
| 290 | ENSG00000239699 | HNRNPA3P8 |  |  | 36240 | NaN | 0.3827 | No |
| 291 | ENSG00000261281 | AC009712.1 |  |  | 36248 | NaN | 0.3826 | No |
| 292 | ENSG00000261284 | RBM22P13 |  |  | 36250 | NaN | 0.3826 | No |
| 293 | ENSG00000228814 | AC017007.1 |  |  | 36349 | NaN | 0.3810 | No |
| 294 | ENSG00000234491 | HNRNPA1P51 |  |  | 36547 | NaN | 0.3777 | No |
| 295 | ENSG00000219039 | AC005102.1 |  |  | 36567 | NaN | 0.3774 | No |
| 296 | novel.687 | - |  |  | 36866 | NaN | 0.3723 | No |
| 297 | ENSG00000255703 | AC127070.3 |  |  | 36990 | NaN | 0.3703 | No |
| 298 | ENSG00000282246 | AL157392.5 |  |  | 36995 | NaN | 0.3702 | No |
| 299 | ENSG00000255051 | BCAS2P1 |  |  | 37413 | NaN | 0.3631 | No |
| 300 | ENSG00000270521 | AL450336.1 |  |  | 37464 | NaN | 0.3623 | No |
| 301 | ENSG00000270526 | SNRPGP19 |  |  | 37467 | NaN | 0.3623 | No |
| 302 | ENSG00000233943 | AC114973.1 |  |  | 37477 | NaN | 0.3622 | No |
| 303 | ENSG00000236459 | HNRNPA1P22 |  |  | 37577 | NaN | 0.3605 | No |
| 304 | ENSG00000236072 | AL592043.2 |  |  | 37598 | NaN | 0.3602 | No |
| 305 | ENSG00000236695 | HNRNPA1P47 |  |  | 37630 | NaN | 0.3597 | No |
| 306 | ENSG00000237207 | RBM17P3 |  |  | 37681 | NaN | 0.3589 | No |
| 307 | ENSG00000259335 | HNRNPMP1 |  |  | 37693 | NaN | 0.3587 | No |
| 308 | ENSG00000258179 | AC079598.3 |  |  | 37730 | NaN | 0.3581 | No |
| 309 | ENSG00000214204 | HNRNPA1P43 |  |  | 37767 | NaN | 0.3576 | No |
| 310 | ENSG00000226789 | AC110926.1 |  |  | 38075 | NaN | 0.3523 | No |
| 311 | ENSG00000226625 | RBM17P1 |  |  | 38445 | NaN | 0.3461 | No |
| 312 | ENSG00000214754 | AC004870.1 |  |  | 38464 | NaN | 0.3458 | No |
| 313 | ENSG00000212961 | HNRNPA1P40 |  |  | 38571 | NaN | 0.3440 | No |
| 314 | novel.597 | - |  |  | 38646 | NaN | 0.3428 | No |
| 315 | ENSG00000248977 | AC095057.1 |  |  | 38798 | NaN | 0.3403 | No |
| 316 | ENSG00000213778 | HNRNPA1P32 |  |  | 38803 | NaN | 0.3402 | No |
| 317 | ENSG00000243188 | HNRNPA1P17 |  |  | 38860 | NaN | 0.3393 | No |
| 318 | ENSG00000233497 | HNRNPA1P60 |  |  | 38945 | NaN | 0.3379 | No |
| 319 | ENSG00000254870 | ATP6V1G2-DDX39B |  |  | 39138 | NaN | 0.3346 | No |
| 320 | ENSG00000205433 | SNRPGP6 |  |  | 39256 | NaN | 0.3327 | No |
| 321 | ENSG00000204560 | DHX16 |  |  | 39481 | NaN | 0.3289 | No |
| 322 | ENSG00000248444 | HNRNPA1P65 |  |  | 39618 | NaN | 0.3266 | No |
| 323 | ENSG00000259550 | HNRNPA1P74 |  |  | 39632 | NaN | 0.3264 | No |
| 324 | ENSG00000224494 | HNRNPA3P14 |  |  | 39932 | NaN | 0.3213 | No |
| 325 | ENSG00000236241 | AC119744.1 |  |  | 40058 | NaN | 0.3192 | No |
| 326 | ENSG00000249920 | HNRNPA1P55 |  |  | 40247 | NaN | 0.3160 | No |
| 327 | ENSG00000227633 | RBMY2YP |  |  | 40299 | NaN | 0.3152 | No |
| 328 | ENSG00000224136 | AC007568.1 |  |  | 40356 | NaN | 0.3143 | No |
| 329 | ENSG00000231206 | HNRNPA1P25 |  |  | 40799 | NaN | 0.3068 | No |
| 330 | ENSG00000226366 | AC097463.1 |  |  | 40834 | NaN | 0.3062 | No |
| 331 | ENSG00000264685 | PRPF19P1 |  |  | 40870 | NaN | 0.3057 | No |
| 332 | ENSG00000275643 | RBM22P6 |  |  | 40950 | NaN | 0.3043 | No |
| 333 | ENSG00000257616 | AC055716.2 |  |  | 41120 | NaN | 0.3015 | No |
| 334 | ENSG00000187999 | HNRNPA1P61 |  |  | 41127 | NaN | 0.3014 | No |
| 335 | ENSG00000257332 | HNRNPA1P69 |  |  | 41200 | NaN | 0.3002 | No |
| 336 | ENSG00000260631 | RBM22P12 |  |  | 41406 | NaN | 0.2967 | No |
| 337 | ENSG00000231137 | RBM22P5 |  |  | 41557 | NaN | 0.2942 | No |
| 338 | ENSG00000214124 | SNRPEP9 |  |  | 41678 | NaN | 0.2922 | No |
| 339 | ENSG00000264186 | SNRPCP4 |  |  | 41742 | NaN | 0.2911 | No |
| 340 | ENSG00000229831 | AC108039.2 |  |  | 41757 | NaN | 0.2909 | No |
| 341 | ENSG00000223429 | AL139156.1 |  |  | 41939 | NaN | 0.2879 | No |
| 342 | ENSG00000278593 | AC104394.1 |  |  | 41947 | NaN | 0.2878 | No |
| 343 | ENSG00000228938 | SNRPD2P1 |  |  | 42184 | NaN | 0.2838 | No |
| 344 | ENSG00000233148 | SYF2P2 |  |  | 42432 | NaN | 0.2796 | No |
| 345 | ENSG00000237804 | HNRNPA1P39 |  |  | 42474 | NaN | 0.2789 | No |
| 346 | ENSG00000255899 | AL662844.2 |  |  | 42673 | NaN | 0.2756 | No |
| 347 | ENSG00000231235 | AL139811.2 |  |  | 42896 | NaN | 0.2718 | No |
| 348 | ENSG00000249271 | HNRNPA1P44 |  |  | 43099 | NaN | 0.2684 | No |
| 349 | ENSG00000259228 | HNRNPA1P62 |  |  | 43208 | NaN | 0.2666 | No |
| 350 | ENSG00000258577 | SNRPGP1 |  |  | 43245 | NaN | 0.2660 | No |
| 351 | ENSG00000229091 | HSPA8P8 |  |  | 43293 | NaN | 0.2653 | No |
| 352 | ENSG00000204392 | LSM2 |  |  | 43399 | NaN | 0.2635 | No |
| 353 | ENSG00000251614 | HSPA8P19 |  |  | 43629 | NaN | 0.2596 | No |
| 354 | ENSG00000236582 | PRPF38AP2 |  |  | 43932 | NaN | 0.2545 | No |
| 355 | ENSG00000250441 | AC109481.1 |  |  | 44009 | NaN | 0.2532 | No |
| 356 | ENSG00000269119 | HNRNPA1P52 |  |  | 44407 | NaN | 0.2465 | No |
| 357 | ENSG00000204390 | HSPA1L |  |  | 44424 | NaN | 0.2462 | No |
| 358 | ENSG00000227453 | HNRNPA1P63 |  |  | 44449 | NaN | 0.2459 | No |
| 359 | ENSG00000179172 | HNRNPCL1 |  |  | 44499 | NaN | 0.2451 | No |
| 360 | ENSG00000253684 | BUD31P1 |  |  | 44508 | NaN | 0.2449 | No |
| 361 | ENSG00000226918 | AC010086.1 |  |  | 44737 | NaN | 0.2411 | No |
| 362 | ENSG00000248926 | AC114801.2 |  |  | 44895 | NaN | 0.2384 | No |
| 363 | ENSG00000237951 | PPIL1P1 |  |  | 45263 | NaN | 0.2322 | No |
| 364 | ENSG00000258350 | SYF2P1 |  |  | 45288 | NaN | 0.2318 | No |
| 365 | ENSG00000259317 | AC044860.3 |  |  | 45307 | NaN | 0.2316 | No |
| 366 | ENSG00000228168 | HNRNPA1P21 |  |  | 45360 | NaN | 0.2307 | No |
| 367 | ENSG00000253605 | HNRNPA1P38 |  |  | 45494 | NaN | 0.2285 | No |
| 368 | ENSG00000250826 | HNRNPA3P13 |  |  | 46099 | NaN | 0.2182 | No |
| 369 | ENSG00000248159 | HSPA8P11 |  |  | 46301 | NaN | 0.2148 | No |
| 370 | ENSG00000233307 | AC011753.3 |  |  | 46347 | NaN | 0.2141 | No |
| 371 | ENSG00000233780 | HNRNPA1P30 |  |  | 46550 | NaN | 0.2106 | No |
| 372 | ENSG00000257539 | HSPA8P14 |  |  | 46651 | NaN | 0.2090 | No |
| 373 | ENSG00000230276 | AL162388.1 |  |  | 46687 | NaN | 0.2084 | No |
| 374 | ENSG00000269576 | HNRNPMP2 |  |  | 46788 | NaN | 0.2067 | No |
| 375 | ENSG00000267178 | PHF5CP |  |  | 47221 | NaN | 0.1994 | No |
| 376 | ENSG00000241947 | HNRNPA1P24 |  |  | 47497 | NaN | 0.1947 | No |
| 377 | ENSG00000265097 | RBM22P1 |  |  | 48505 | NaN | 0.1776 | No |
| 378 | ENSG00000214144 | AL390728.1 |  |  | 49143 | NaN | 0.1668 | No |
| 379 | ENSG00000224059 | HSPA8P16 |  |  | 49235 | NaN | 0.1652 | No |
| 380 | ENSG00000233680 | HNRNPA1P27 |  |  | 49532 | NaN | 0.1602 | No |
| 381 | ENSG00000218454 | HNRNPA1P19 |  |  | 49536 | NaN | 0.1602 | No |
| 382 | ENSG00000267233 | HNRNPA3P16 |  |  | 49588 | NaN | 0.1594 | No |
| 383 | ENSG00000229668 | AL133384.1 |  |  | 49611 | NaN | 0.1590 | No |
| 384 | ENSG00000257847 | LSM3P2 |  |  | 49770 | NaN | 0.1563 | No |
| 385 | ENSG00000235864 | HSPA8P6 |  |  | 49875 | NaN | 0.1546 | No |
| 386 | ENSG00000216835 | RBMXP1 |  |  | 50268 | NaN | 0.1479 | No |
| 387 | ENSG00000217824 | SNRPEP6 |  |  | 50361 | NaN | 0.1464 | No |
| 388 | ENSG00000238001 | SNRPCP1 |  |  | 50375 | NaN | 0.1462 | No |
| 389 | ENSG00000228551 | SNRPGP9 |  |  | 50431 | NaN | 0.1453 | No |
| 390 | ENSG00000228557 | HSPA8P17 |  |  | 50433 | NaN | 0.1453 | No |
| 391 | ENSG00000242915 | SNRPGP7 |  |  | 50449 | NaN | 0.1451 | No |
| 392 | ENSG00000254425 | AC022690.1 |  |  | 50452 | NaN | 0.1451 | No |
| 393 | ENSG00000235681 | AC245884.3 |  |  | 50545 | NaN | 0.1435 | No |
| 394 | ENSG00000277769 | RBM22P4 |  |  | 50581 | NaN | 0.1430 | No |
| 395 | ENSG00000271338 | AL591034.2 |  |  | 50611 | NaN | 0.1425 | No |
| 396 | ENSG00000224314 | AC005002.1 |  |  | 50645 | NaN | 0.1420 | No |
| 397 | ENSG00000220541 | AC233289.1 |  |  | 50684 | NaN | 0.1413 | No |
| 398 | ENSG00000227002 | SNRPEP10 |  |  | 50713 | NaN | 0.1409 | No |
| 399 | ENSG00000238024 | DDX39BP2 |  |  | 50771 | NaN | 0.1400 | No |
| 400 | ENSG00000215054 | HNRNPCP10 |  |  | 50850 | NaN | 0.1387 | No |
| 401 | ENSG00000231480 | SNRPGP13 |  |  | 50918 | NaN | 0.1375 | No |
| 402 | ENSG00000261796 | ISY1-RAB43 |  |  | 51046 | NaN | 0.1354 | No |
| 403 | ENSG00000226188 | HNRNPA1P3 |  |  | 51075 | NaN | 0.1349 | No |
| 404 | ENSG00000241804 | AC126121.1 |  |  | 51324 | NaN | 0.1308 | No |
| 405 | ENSG00000258271 | AC025260.1 |  |  | 51380 | NaN | 0.1298 | No |
| 406 | ENSG00000258856 | AL136520.1 |  |  | 51406 | NaN | 0.1294 | No |
| 407 | ENSG00000227624 | SNRPEP3 |  |  | 51471 | NaN | 0.1284 | No |
| 408 | ENSG00000259464 | SNRPCP18 |  |  | 51742 | NaN | 0.1238 | No |
| 409 | ENSG00000205412 | HNRNPA1P20 |  |  | 52178 | NaN | 0.1164 | No |
| 410 | ENSG00000260389 | WBP11P1 |  |  | 52179 | NaN | 0.1164 | No |
| 411 | ENSG00000270612 | AC112191.3 |  |  | 52446 | NaN | 0.1119 | No |
| 412 | novel.654 | - |  |  | 52539 | NaN | 0.1104 | No |
| 413 | ENSG00000264315 | HNRNPA1P11 |  |  | 52653 | NaN | 0.1085 | No |
| 414 | ENSG00000270494 | AL513534.2 |  |  | 52975 | NaN | 0.1031 | No |
| 415 | ENSG00000258073 | AC128657.1 |  |  | 53067 | NaN | 0.1015 | No |
| 416 | ENSG00000227965 | AC098592.2 |  |  | 53189 | NaN | 0.0995 | No |
| 417 | ENSG00000234399 | RBMY2XP |  |  | 53531 | NaN | 0.0937 | No |
| 418 | ENSG00000235400 | AC104837.1 |  |  | 53740 | NaN | 0.0902 | No |
| 419 | ENSG00000218347 | HNRNPA1P1 |  |  | 53853 | NaN | 0.0883 | No |
| 420 | ENSG00000213126 | AC092642.1 |  |  | 54033 | NaN | 0.0853 | No |
| 421 | ENSG00000236946 | HNRNPA1P70 |  |  | 54163 | NaN | 0.0831 | No |
| 422 | ENSG00000269014 | AC008622.1 |  |  | 54553 | NaN | 0.0765 | No |
| 423 | ENSG00000225053 | PRPF38AP1 |  |  | 54590 | NaN | 0.0759 | No |
| 424 | ENSG00000270962 | AC239800.4 |  |  | 54750 | NaN | 0.0733 | No |
| 425 | ENSG00000260014 | LSM3P5 |  |  | 54901 | NaN | 0.0707 | No |
| 426 | ENSG00000213568 | HNRNPA1P13 |  |  | 55034 | NaN | 0.0685 | No |
| 427 | ENSG00000230785 | AC006326.1 |  |  | 55129 | NaN | 0.0669 | No |
| 428 | ENSG00000260675 | AC109446.2 |  |  | 55266 | NaN | 0.0646 | No |
| 429 | ENSG00000218574 | HNRNPA1P37 |  |  | 55352 | NaN | 0.0632 | No |
| 430 | ENSG00000213365 | AP000593.1 |  |  | 55387 | NaN | 0.0627 | No |
| 431 | ENSG00000226718 | SNRPGP8 |  |  | 55420 | NaN | 0.0622 | No |
| 432 | ENSG00000170748 | RBMXL2 |  |  | 55490 | NaN | 0.0610 | No |
| 433 | ENSG00000175718 | RBMXL3 |  |  | 55519 | NaN | 0.0606 | No |
| 434 | ENSG00000240236 | HNRNPA1P23 |  |  | 55814 | NaN | 0.0556 | No |
| 435 | ENSG00000234176 | HSPA8P1 |  |  | 55913 | NaN | 0.0539 | No |
| 436 | ENSG00000213049 | HNRNPA1P34 |  |  | 56001 | NaN | 0.0525 | No |
| 437 | ENSG00000258988 | AL135978.1 |  |  | 56006 | NaN | 0.0524 | No |
| 438 | ENSG00000255287 | SNRPGP16 |  |  | 56167 | NaN | 0.0497 | No |
| 439 | ENSG00000248891 | AC091860.1 |  |  | 56288 | NaN | 0.0477 | No |
| 440 | ENSG00000229534 | HNRNPA1P53 |  |  | 56408 | NaN | 0.0457 | No |
| 441 | ENSG00000270665 | HNRNPA1P67 |  |  | 56961 | NaN | 0.0363 | No |
| 442 | ENSG00000235784 | HNRNPA1P29 |  |  | 56981 | NaN | 0.0360 | No |
| 443 | ENSG00000229115 | AL390774.1 |  |  | 57099 | NaN | 0.0341 | No |
| 444 | ENSG00000228051 | AL512504.2 |  |  | 57345 | NaN | 0.0299 | No |
| 445 | ENSG00000271671 | AC107304.1 |  |  | 57563 | NaN | 0.0263 | No |
| 446 | ENSG00000275826 | AC093909.5 |  |  | 57613 | NaN | 0.0254 | No |
| 447 | ENSG00000224640 | SRSF1P1 |  |  | 57719 | NaN | 0.0237 | No |
| 448 | ENSG00000227688 | HNRNPA3P2 |  |  | 57821 | NaN | 0.0220 | No |
| 449 | ENSG00000229221 | HNRNPA1P66 |  |  | 57833 | NaN | 0.0218 | No |
| 450 | ENSG00000275862 | AC008622.3 |  |  | 58414 | NaN | 0.0120 | No |
| 451 | ENSG00000219582 | HNRNPA1P58 |  |  | 58660 | NaN | 0.0078 | No |
| 452 | ENSG00000229110 | AC006355.1 |  |  | 58799 | NaN | 0.0055 | No |
| 453 | ENSG00000259686 | HNRNPA1P71 |  |  | 58836 | NaN | 0.0049 | No |
| 454 | ENSG00000225990 | SNRPEP7 |  |  | 58853 | NaN | 0.0047 | No |
| 455 | ENSG00000276841 | AL445264.1 |  |  | 58875 | NaN | 0.0043 | No |
| 456 | ENSG00000233615 | HNRNPA1P42 |  |  | 58923 | NaN | 0.0036 | No |
| 457 | ENSG00000231956 | HNRNPA1P9 |  |  | 59006 | NaN | 0.0022 | No |
Table: GSEA details [plain text format]

  

Fig 2: SPLICEOSOME(HSA03040)      
 Blue-Pink O' Gram in the Space of the Analyzed GeneSet

  

Fig 3: SPLICEOSOME(HSA03040): Random ES distribution      
 Gene set null distribution of ES for **SPLICEOSOME(HSA03040)**
